# There Is No ‘Rule of Thumb’: Genomic Filter Settings for a Small Plant Population to Obtain Unbiased Gene Flow Estimates

**DOI:** 10.3389/fpls.2021.677009

**Published:** 2021-10-14

**Authors:** Alison G. Nazareno, L. Lacey Knowles

**Affiliations:** ^1^Department of Ecology and Evolutionary Biology, University of Michigan, Ann Arbor, MI, United States; ^2^Department of Genetics, Ecology and Evolution, Federal University of Minas Gerais, Belo Horizonte, Brazil

**Keywords:** conservation genetics, *Dinizia jueirana-facao*, Fabaceae, spatial genetic structure, parentage assignment

## Abstract

The application of high-density polymorphic single-nucleotide polymorphisms (SNP) markers derived from high-throughput sequencing methods has heralded plenty of biological questions about the linkages of processes operating at micro- and macroevolutionary scales. However, the effects of SNP filtering practices on population genetic inference have received much less attention. By performing sensitivity analyses, we empirically investigated how decisions about the percentage of missing data (MD) and the minor allele frequency (MAF) set in bioinformatic processing of genomic data affect direct (i.e., parentage analysis) and indirect (i.e., fine-scale spatial genetic structure – SGS) gene flow estimates. We focus specifically on these manifestations in small plant populations, and particularly, in the rare tropical plant species *Dinizia jueirana-facao*, where assumptions implicit to analytical procedures for accurate estimates of gene flow may not hold. Avoiding biases in dispersal estimates are essential given this species is facing extinction risks due to habitat loss, and so we also investigate the effects of forest fragmentation on the accuracy of dispersal estimates under different filtering criteria by testing for recent decrease in the scale of gene flow. Our sensitivity analyses demonstrate that gene flow estimates are robust to different setting of MAF (0.05–0.35) and MD (0–20%). Comparing the direct and indirect estimates of dispersal, we find that contemporary estimates of gene dispersal distance (σ*_r_*_t_ = 41.8 m) was ∼ fourfold smaller than the historical estimates, supporting the hypothesis of a temporal shift in the scale of gene flow in *D. jueirana-facao*, which is consistent with predictions based on recent, dramatic forest fragmentation process. While we identified settings for filtering genomic data to avoid biases in gene flow estimates, we stress that there is no ‘rule of thumb’ for bioinformatic filtering and that relying on default program settings is not advisable. Instead, we suggest that the approach implemented here be applied independently in each separate empirical study to confirm appropriate settings to obtain unbiased population genetics estimates.

## Introduction

High-throughput sequencing technologies that take advantage of restriction endonuclease enzymes to generate reduced representations of genomes (Davey et al., 2011; Andrews et al., 2016) are enabling us to identify, sequence, and genotype thousands of SNPs (i.e., single-nucleotide polymorphisms) in any kind of organism. This use of high-density biallelic SNP markers has heralded a plethora of evolutionary questions at a genome-level in non-model organisms, improving our understanding of the underlying processes at micro- and macroevolutionary scales (Alencar and Quental, 2019; Myers et al., 2019). Furthermore, the increasing number and density of molecular markers across the genome can give more statistical power for accurate population genetic parameters (e.g., Luikart et al., 2003; Nazareno et al., 2017). This becomes invaluable for addressing questions where the processes of interest act locally, and hence at finer spatial and temporal scales, rather than at large spatial or temporal scales. For example, the negative effects of habitat fragmentation often manifest at local scales, especially in organisms with limited dispersal capabilities. As such, the effect of fragmentation may only be detectable with the resolution of genomic data. For example, analysis of hundreds of SNPs in an endangered salamander revealed the effects of fragmentation on genetic diversity and structure (McCartney-Melstad et al., 2018), but such effects went undetected in analyses of few microsatellite markers (Titus et al., 2014).

With time- and cost-efficient techniques and lower genotyping error than other molecular markers (e.g., microsatellites) (e.g., Davey et al., 2011; Seeb et al., 2011; O’Leary et al., 2018), the sharp rise in applications of SNPs in population genetic studies (Andrews et al., 2016) has also been accompanied by many studies on best practices. These include details ranging from library preparation to bioinformatic processing for quality controls to improve the accuracy of SNP data sets (e.g., DePristo et al., 2011; Gautier et al., 2013; Ilut et al., 2014; Mastretta-Yanes et al., 2015; Andrews et al., 2016; Paris et al., 2017; Willis et al., 2017; O’Leary et al., 2018; Díaz-Arce and Rodríguez-Ezpeleta, 2019; Cumer et al., 2021). However, the effects of some SNP filtering practices, especially in relation to parameters regarding the frequency of missing data (MD) and minor allele frequency (MAF), and their effects on population genetic inference have received much less attention (Huang and Knowles, 2014; Andrews et al., 2018; Hall et al., 2020). Some questions, especially those focused on local spatial and temporal scales, are no doubt disproportionately affected by these filtering practices. Ironically, these are also scenarios where the large number of SNPs are required for distinguishing among hypotheses, where such distinction rests on subtle differences in the allele frequency spectrum, and yet, this spectrum is sensitive to filtering practices (see Huang and Knowles, 2014), biasing parameter estimates (Larson et al., 2020).

Here we address the unintended consequences of filtering practices of RADseq on estimates of dispersal, focusing on the MD and MAF settings used in bioinformatic processing. Specifically, we examine the effects of filtering on dispersal estimates for an endangered species threatened with extinction, *Dinizia jueirana-facao* G. P. Lewis and G. S. Siqueira (Fabaceae: Caesalpinioideae), where accurate parameter estimation has downstream consequences for conservation decisions; conservation concerns were the primary motivation for the collection of genetic data in this species. This recently discovered species (Lewis et al., 2017) is facing extinction risks due to habitat fragmentation and degradation. With notable reductions in populations, the species has become increasingly rare, with a few remaining small populations. Consequently, both direct measures of dispersal from parentage analyses, as well as indirect estimates of dispersal from fine-scale spatial genetic structure (SGS; i.e., non-random spatial distribution of genotypes within populations), provide useful information for management activities and policies, including seed collection for *ex situ* conservation, tree breeding, and/or reforestation (e.g., Bittencourt and Sebbenn, 2007; Ramos et al., 2018; de Oliveira et al., 2020).

While the effects of MD and MAF used in bioinformatic processing of SNP data on fine-scale SGS (Attard et al., 2018) employing different relatedness coefficients (e.g., Loiselle et al., 1995; Ritland, 1996, kinship estimators; Queller and Goodnight, 1989) have been investigated more generally (see Hellmann et al., 2016; de Fraga et al., 2017; Escoda et al., 2017; Attard et al., 2018), the results (and therefore recommendations for best practices) from such studies may not be generalizable to small populations for a number of reasons. For example, studies have shown that parentage assignments are accurate with high statistical power when the frequency of both alleles is close to 0.5 (Anderson and Garza, 2006; Baruch and Weller, 2008; Strucken et al., 2016; Andrews et al., 2018; Dussault and Boulding, 2018; but see Andrews et al., 2018) or when there is no missing data in the SNP data set (see Dussault and Boulding, 2018). However, such conditions are unlikely to be met when studying threatened species. Such taxa generally have low genetic variation and their populations are often comprised of closely related individuals.

By informing our study through the analysis of empirical data, we assure that the observed effects of MAF and MD settings when processing genomic data are consistent with the biological realities of being a rare, endangered plant species. We follow our analyses of the endangered plant species *D. jueirana-facao* with a discussion of why different MD and MAF settings among taxa are likely necessary to obtain unbiased estimates of dispersal, as opposed to general guidelines about MD and MAF that do not consider specific applications of RADseq data (e.g., Andrews et al., 2018; Dussault and Boulding, 2018). By comparing the direct and indirect gene flow estimates, we also investigate the effects of forest fragmentation by testing the hypothesis of recent decrease in the scale of gene flow expressed by the dispersal distance. This pattern is expected because only direct estimates should be affected due to the temporal inertia of indirect estimates for few generations (Dutech et al., 2005; Oddou-Muratorio and Klein, 2008). Lastly, evaluation of the sensitivity of both direct and indirect estimates of gene flow to settings of MD and MAF for SNP data sets shows that the assumptions of general bioinformatic guidelines are not likely to be met in species that have recently undergone declines and/or are rare.

## Materials and Methods

### Focal Taxon, Study Area and Sampling

**FIGURE 1:**
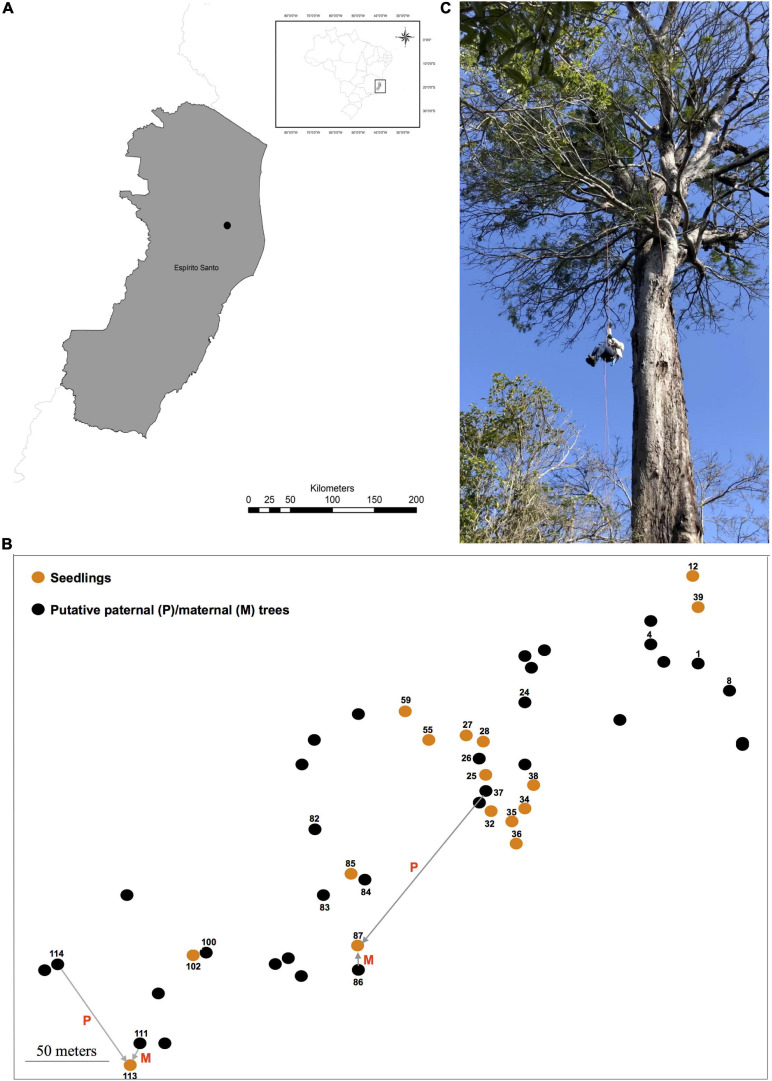
The location **(A)** of the studied population (black dot) in the Atlantic Forest (Reserva Natural Vale, Espírito Santo State, Southeast Brazil). The spatial distribution of reproductive trees (i.e., putative maternal and/or paternal parents) and seedlings of *Dinizia jueirana-facao* sampled in the small population is shown in **(B)**. The reproductive trees (black dots) that were assigned as a maternal and/or paternal parent of the seedlings (orange dots) are also shown in **(B)** (displayed numbers; see Table 3 to identify the maternal and/or paternal parents of the seedlings as shown for seedlings 87 and 113). An image of an adult tree **(C)** is also shown.

*Dinizia jueirana-facao* is a narrowly restricted tree species endemic to a small area of the Brazilian Atlantic Forest (Figure 1). The inflorescences of *D. jueirana-facao* are composed of hermaphrodite yellow flowers, with some apical flowers appearing functionally male due to suppression of gynoecium development (Lewis et al., 2017), and its scimitar-shaped and woody large fruits (40–46 × 8.5–10 cm) contain black and hard seeds (Lewis et al., 2017). Although there is a morphological characterization of the reproductive structures of *D. jueirana-facao* (Lewis et al., 2017), there is no information about how its pollen and seeds are dispersed to date. This canopy-emergent tree (19–40 m) is Critically Endangered due to ongoing decline in the number of adult trees because of habitat deforestation and occurs in only two localities: one within the Reserva Natural Vale (RNV) and the other ca. 12.0 km away from the reserve. Only 12 adult trees at RNV, and another 12 trees, were previously mapped in these restricted areas, which combined cover a little over 100 hectares (ha) (Lewis et al., 2017). Fortunately, after an intense sampling effort in 2019, we were able to expand this sampling for the species in the RNV to include 16 seedlings (H, Height, <61 cm) and 99 trees (DBH, Diameter at Breast Height, >10.0 cm; H > 4.0 m), 34 of which were reproductive (DBH > 87 cm, H > 9.0 m). We also confirmed the number of individuals that reproduced in the observed event plus those that had reproduced in a previous event based on the presence of dried reproductive pods and/or seeds under the plant.

For the genetic study, leaf samples of reproductive trees (34 plants with H varying from 9 to 30 m and with a DBH ranging from 87 to 490 cm) and 16 seedlings (H varying from 20 to 60 cm) closest to a reproductive tree were collected and mapped (Figure 1) within the RNV site. The distances between adult trees ranged from 2.36 to 444.80 m (average 128.00 ± 60.67 m), and the distances between seedlings ranged from 2.90 to 375.40 m (average 112.16 ± 84.05 m).

### Library Preparation and Sequencing

We extracted genomic DNA from leaf samples of 50 individuals using the Macherey-Nagel kit (Macherey-Nagel GmbH & Co. KG), following the manufacturer’s instructions. We created one genomic library using a double-digest restriction site-associated DNA sequencing (i.e., ddRADseq) protocol (Peterson et al., 2012), with modifications to minimize the risk of high variance in the number of reads per individual (see Nazareno et al., 2017 for more details). Briefly, PCRs were performed on each individual and amplicons were pooled for size selection, instead of pooling samples prior to PCR as recommended by Peterson et al. (2012). Double-stranded DNA concentrations were quantified using the Qubit dsDNA Assay Kit (Invitrogen) and 0.5 μg for each individual was digested with the high-fidelity restriction enzymes *Eco*RI and *Mse*I (New England Biolabs). Digestion reactions were purified with the Agencourt AMPure XP system (Beckman Coulter), following the manufacturer’s instructions, with elution in 40 μL water. Adapter ligations were carried out at 23°C for 30 min in a total volume of 30 μL, combining 80 ng of DNA, 0.35 μM of a non-sample specific *Mse*I adaptor (common for all samples), 0.50 μM of a sample specific *Eco*RI double-strand adaptor for each DNA sample, 1U of T4 DNA ligase (New England BioLabs), and 1.5 × T4 ligase buffer. Reactions were heated at 65°C for 10 min and slowly cooled to 23°C. Ligation products were cleaned with the Agencourt AMPure XP system and amplified following the PCR protocol reported by Nazareno et al. (2017). Multiplexed genomic library was prepared with approximately equal amounts of DNA, and DNA fragments at a target range size of 375–475 bp were size-selected using Pippin Prep and a 2% agarose cartridge (Sage Science, Beverly, MA, United States). The library was sequenced (100 bp single-end reads) on a lane of an Illumina HiSeq 2500 flowcell (Illumina Inc., San Diego, CA, United States) at The Centre for Applied Genomics in Toronto, Canada.

### SNPs Identification

Files containing the raw sequence reads were analyzed in Stacks 2.41 (Catchen et al., 2011; Catchen et al., 2013; Rochette et al., 2019) using *de novo* assembly. We used the process_radtags program in Stacks to initially assign reads to individuals and eliminate poor quality reads and reads missing the expected *Eco*RI cut site (options –barcode_dist 2 -q -e ecoRI). All sequences were processed in ustacks to produce consensus sequences of RAD tags, applying a maximum-likelihood framework to estimate the diploid genotype for each individual at each nucleotide position (Hohenlohe et al., 2011). The optimum minimum depth of coverage to create a stack was set at three sequences, the maximum distance allowed between stacks was two nucleotides, and the maximum number of stacks allowed per *de novo* locus was three. The stacks assembly enabled the Deleveraging algorithm (–d), which resolves over-merged tags, and the Removal algorithm (–r), which drops highly repetitive stacks and nearby errors from the algorithm. The alpha value for the SNP model was set at 0.05; as reported by Catchen et al. (2013); low alpha values (i.e., <0.10) avoid underestimating true heterozygous genotypes. Cstacks was used to build a catalog of consensus loci containing all the loci from all the individuals and merging all alleles together. After processing the consensus loci in cstacks, stacks generated were searched against the catalog and SNPs were called using sstacks, tsv2bam, and gstacks (Rochette et al., 2019), with default settings.

### Data Sets With Different Amounts of Missing Data (MD) and Minor Allele Frequency (MAF)

We used POPULATIONS in Stacks (Catchen et al., 2011, 2013; Rochette et al., 2019) to create data sets with five different MD settings (i.e., 0, 5, 10, 15, and 20%) and seven different MAF settings (i.e., 0.05, 0.10, 0.15, 0.20, 0.25, 0.30, and 0.35), for a total of 35 data sets. Note, it was not possible to generate datasets with MD > 20% and MAF > 0.35 because the combination of such parameter settings resulted in very small number of SNPs in our empirical data sets. All data sets include one SNP per locus, which were identified after filtering loci to confirm Hardy--Weinberg (H-W) equilibrium and linkage disequilibrium (LD) using the adegenet package^1^ (Jombart, 2008; Jombart and Ahmed, 2011) implemented in R (R Core Team, 2018) and Arlequin 3.5.2 (Excoffier and Lischer, 2010), respectively. Type I error rates for these tests were corrected for multiple tests using the sequential Bonferroni procedure (Rice, 1989) and SNPs that failed the H-W equilibrium test and/or SNP pairs in LD were excluded.

### Assessing the Effects of MD and MAF on SGS and Indirect Dispersal Distance

Assuming that genotypes of all *D. jueirana-facao* come from a two-dimensional population at drift-dispersal equilibrium, the spatial genetic structure (SGS) was inferred based on pairwise relatedness coefficients between individuals using the SPAGeDi program (Hardy and Vekemans, 2002). Estimators of kinship (co-ancestry) coefficients (*F*_ij_) and relationship coefficients (*R*_ij_) were calculated for different groups of individuals, where the groups differ by the geographic distance separating them. Specifically, the kinship coefficient estimators *F*_L_ (Loiselle et al., 1995) and *F*_r_ (Ritland, 1996) are based on the probability that a random allele from individual *i* is identical by descent to a random allele from individual *j* (Vekemans and Hardy, 2004); *F*_r_ is downward biased when very low frequency alleles occur (Ritland, 1996; Vekemans and Hardy, 2004). The *R*_Q&G_ estimator (Queller and Goodnight, 1989) is based on the probability that a random allele from individual *i* is identical to one of the alleles from individual *j* (Vekemans and Hardy, 2004). Although the estimators display high discriminate power in allozymes and microsatellites markers (Vekemans and Hardy, 2004), there is limited information on their performance with SNP markers (e.g., Attard et al., 2018), especially for SNPs collected in small populations. In addition to the close relation between *Fij* and *Rij*, the *F*_L_, *F*_r_, and *R*_Q&G_ estimators can be used in a comparative way (for a more detailed account on statistical properties of these estimators see Queller and Goodnight, 1989; Loiselle et al., 1995; Ritland, 1996), given some estimators make no assumption regarding Wright’s inbreeding coefficient (i.e., the probability that two randomly chosen alleles of an individual at any homologous locus are identical by descent; Malécot, 1948). Individuals were grouped into six distance classes to maximize the number of pairs of individuals per distance class and the average multi-locus relatedness coefficients [*F*_(d)_ or *R*_(d)_] per distance class using the SPAGeDi program (Hardy and Vekemans, 2002). The 95% confidence interval (CI) of the standard error of the relatedness coefficients was calculated using a jackknife procedure across all loci.

In order to test for significant SGS, *F*_ij_ (or *R*_ij_), all pairs of individuals were plotted against the log pairwise spatial distance and significance of the regression was assessed by 10,000 permutations of multilocus genotypes. To compare the extent that SGS varies among data sets with different MD- and MAF-values, we calculated the *Sp*-statistic, a synthetic measure of SGS intensity that is less sensitive to the sampling scheme, and that expresses the balance between local genetic drift and gene dispersal within population (Vekemans and Hardy, 2004; Hardy et al., 2006). The *Sp-*statistic is defined as: *Sp* = -*b*/(1-*F*_1_), where *b* is the regression slope of *F*_ij_ on log spatial distance, and *F*_1_ is the mean *F*_ij_ between individuals for the first distance class (Vekemans and Hardy, 2004); the parameters to calculate the *Sp-*statistic were obtained in SPAGeDi, and to compute *Sp* using the *R*_Q&G_ estimator, we converted the *R*_ij_ values in *F*_ij_ applying the equation *R*_ij_ = 2*F*_ij_/(1 + *F*_I_) as proposed by Hardy and Vekemans (1999).

To investigate the relative sensitivity of the different relatedness estimators to the MD and MAF, we used the mean *F*_ij_ and the *Sp*-statistic values computed considering all the sampled individuals (*n* = 50). Following this result, we used the estimator with the minimum relative standard deviation (i.e., coefficient of variation) to assess the effects of MD and MAF on SGS estimates. As there is a direct association between co-ancestry and inbreeding (i.e., in generation *T*_1_ the inbreeding coefficient is equal the co-ancestry in generation *T*_0_; Cockerham, 1966), we also investigated the effects of MD and MAF on Wright’s inbreeding coefficient. These analyses were performed separately for the reproductive trees (*n* = 34) and seedlings (*n* = 16) of *D. jueirana-facao.*

Lastly, the root-mean-squared dispersal distance (σ) was calculated using the *Sp*-statistic and the effective population density *D*_e_, which is the product of the census density *D* and *N_e_/N*, the ratio of the effective to the census population size, where σ *^2^* = *N_b_/4* π *D*_e_ (Vekemans and Hardy, 2004). While *D*_e_ in plant populations can be estimated as *D/*4 (Hardy et al., 2006), the *D*-values of reproductive trees (0.79 ind.ha^–1^) and seedlings (0.37 ind.ha^–1^), were multiplied by 0.30 – the *N_e_/N* ratio directly computed for the *D. jueirana-facao* in the RNV site given that the Wright’s neighborhood size (*N*_b_) equals 1/*Sp* (Vekemans and Hardy, 2004). The *N_e_/N* ratio in the RNV population (i.e., 0.30) was calculated as the number of reproductive trees (*N_e_* = 34) divided by the total number of *D. jueirana-facao* plants [*N* = 115; i.e., adult trees (*n* = 99) + seedlings (*n* = 16)].

### Assessing the Effects of MD and MAF on Parentage Analysis

We used the CERVUS 3.0.7 program (Marshall et al., 1998; Kalinowski et al., 2007) to investigate how MD and MAF affect cryptic gene flow, *C*_gf_, which expresses the proportion of genotypes assigned to a candidate parent within the sampled area when the true parent is located outside there. *C*_gf_ was calculated as 1 – (1 – *P*_p_)*^n^*, where *n* is the number of candidate parents within the population, and *P*_p_ represents the combined non-exclusion probability (i.e., the probability of not excluding a single randomly chosen unrelated individual from parentage over all loci) of the parent pair, when the parent pair is unknown (Dow and Ashley, 1996). Then, we identified the appropriate data set (i.e., those with *C*_gf_ ≈ zero) to be used on the characterization of direct gene flow in the *D. jueirana-facao* population following the categorical parentage analyses described below.

Parentage analyses were performed according to the maximum likelihood method integrated in the CERVUS (Marshall et al., 1998; Kalinowski et al., 2007), with 50,000 simulated genotypes to estimate the critical value of Delta (Δ*_crit_*), considering a genotyping error ratio of 1%. The proportion of candidate parents sampled was set at 90%, which was justifiable given that we had genotyped all known adult trees of *D. jueirana-facao* in the RNV population, which takes into account that 10% of candidate parents may have died in recent years. Parentage assignment was performed comparing the value of Δ*_crit_* with the Δ-score. The Δ-score is used as a criterion for the assignment of parentage and it is defined as the difference in LOD scores between the most likely candidate parent and the second most likely candidate parent. As defined by Marshall et al. (1998), the LOD score is the natural logarithm of the likelihood that the candidate parent is the true parent divided by the likelihood that the candidate parent is not the true parent. Gene flow via pollen and seed dispersal was estimated in seedlings (*n* = 16) considering all reproductive trees (*n* = 34) as possible maternal and/or paternal candidates. Putative parents were recognized as those with Δ > Δ*_crit_* with 95% confidence; seedlings in which the same tree was inferred to be the maternal and paternal parent were considered to represent examples of selfing. Pollen and seed dispersal (Euclidean) distances were calculated considering the distance between seedling and the putative parents. Specifically, seedlings with only one putative parent identified within the population were presumed to represent the distance of seed dispersal, assuming that pollen dispersal distances are more likely to come from geographically more distant reproductive trees that were not sampled (Dow and Ashley, 1996). For seedlings with two putative parents identified among the reproductive trees of the study population, the one nearest to the seedling was presumed to reflect seed dispersal (i.e., the maternal parent), whereas the more distant one was presumed to reflect pollen dispersal (i.e., the paternal parent), again using the assumption that the distance traveled by pollen is likely greater than that of seeds, which has been applied in parentage analyses when the maternal parent is not distinguishable (e.g., Dow and Ashley, 1996; Guidugli et al., 2016; Feres et al., 2021), as is the case of the hermaphroditic tree species such as *D. jueirana-facao*. We also calculated seed (*m*_s_) and pollen (*m*_p_) immigration rates. Specifically, immigrant seeds and immigrant pollen were represented by seedlings without assigned parents or seedlings that had only one putative parent assigned from the population, respectively, and were compared relative to the total number of seedlings to discern *m*_s_ and *m*_p_ (Burczyk et al., 1996).

Lastly, for comparison with the root-mean-squared dispersal distance obtained from the SGS analysis, we computed the total direct gene flow (σ*^2^_rt_*) using the parentage assignment results. Specifically, the total direct gene flow, σ*^2^_rt_* is equal to 1/2 σ*^2^_p–rt_* + σ*^2^_s–rt_*, where σ*^2^_p–rt_* and σ*^2^_s–rt_* are the variances of the pollen and seed dispersal distances, respectively (Crawford, 1984).

## Results

About 145 million single-end raw reads were produced on one sequencing lane of HiSeq 2000 Illumina for the 50 individuals included in the genomic library of *D. jueirana-facao*. The mean number of retained reads that passed the quality filters, including a Phred quality score > 33 with identifiable barcodes, were 2,319,619 ± 152,193 SE. The number of polymorphic SNPs with a minimum 10-fold coverage ranged from 256 (MD = 0%, MAF = 0.35) to 6,898 (MD = 20%, MAF = 0.05). No significant departures from HWE were observed in any data set after a Bonferroni adjustment (*p* > 0.00019). In addition, no LD was observed after a sequential Bonferroni correction for *k* tests (varying from *k* = 3.26 × 10^4^ with *p* < 1.53 × 10^–6^ for 256 SNPs to *k* = 2.38 × 10^7^ with *p* < 2.10 × 10^–9^ for 6,898 SNPs).

### Effects of MD and MAF on SGS and Indirect Dispersal Distance

**FIGURE 2:**
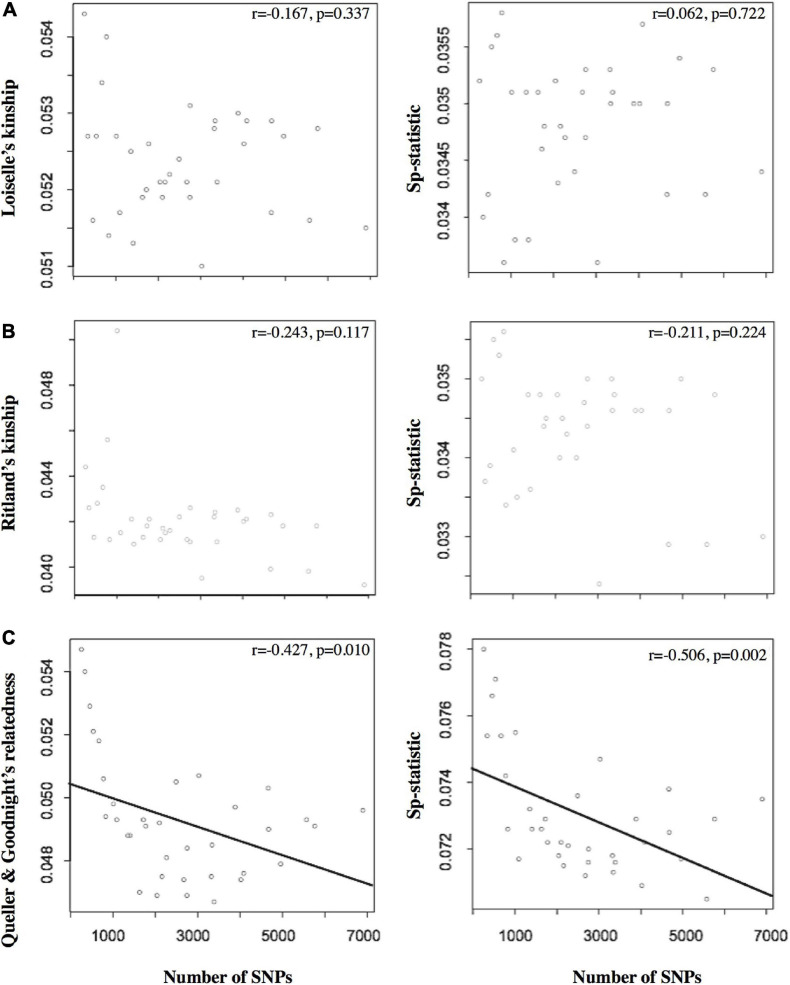
The left column presents the relationship between the number of SNPs and the relatedness estimators based on **(A)** Loiselle’s kinship (Loiselle et al., 1995), **(B)** Ritland’s kinship (Ritland, 1996), and **(C)** Queller and Goodnight’s relatedness. The right column shows the relationship between the number of SNPs and the *Sp*-statistic. Note that the number of SNPs differed as a function of the 35 variable MD/MAF data sets (saw as points in the plots) used to filter the SNP data sets during bioinformatic processing. The Pearson’s correlation coefficient (*r*) and significance are also shown for each plot.

Of the different estimators of relatedness, the Loisele’s kinship estimator was comparatively less sensitive to the different settings of MD and MAF (coefficient of variation, *CV* = 1.37%; Table 1), as well as derivatives based on the relatedness estimator – namely, the measure of the extent of SGS captured by the *Sp-*statistic (*CV* = 1.69%; Table 1). Although the standard error of all estimators of relatedness increases when the number of loci decreases (Supplementary Table 1), the Loisele’s kinship and its derivative *Sp-*statistic were both less sensitive to the number of SNPs analyzed than other relatedness measures, showing a weak, non-significant correlation (Figure 2). Therefore, we based tests of fine-scale SGS on the Loisele’s kinship (see below).

**TABLE 1:** Effects of different amounts of missing data (MD) and minor allele frequency (MAF) on the estimates of pairwise relatedness statistics expressed by the mean *F*_ij_ between individuals for the first distance class (*F*_1_) for the empirical data set (*n* = 50; adults and seedlings of *Dinizia jueirana-facao* combined).

		Loiselle’s kinship				Ritland’s kinship				Queller and Goodnight’s relatedness			
			
MD_MAF	SNPs	*F*_1_ (<50 m)	*b*	*R* ^2^	*Sp*	*F*_1_ (<50 m)	*b*	*R* ^2^	*Sp*	*F*_1_ (<50m)*	*b*	*R* ^2^	*Sp*
1.00_0.35	256	**0.054**	**0.033**	0.187	0.0352	**0.044**	**0.033**	0.188	0.0350	**0.055**	**0.074**	0.192	0.0780
1.00_0.30	338	**0.053**	**0.032**	0.191	0.0340	**0.043**	**0.032**	0.190	0.0337	**0.054**	**0.071**	0.197	0.0754
1.00_0.25	456	**0.052**	**0.032**	0.205	0.0342	**0.041**	**0.032**	0.205	0.0339	**0.053**	**0.073**	0.216	0.0766
1.00_0.20	537	**0.053**	**0.034**	0.217	0.0355	**0.043**	**0.034**	0.220	0.0355	**0.052**	**0.073**	0.218	0.0771
1.00_0.15	668	**0.053**	**0.034**	0.217	0.0356	**0.044**	**0.034**	0.215	0.0353	**0.052**	**0.072**	0.212	0.0754
1.00_0.10	778	**0.054**	**0.034**	0.218	0.0358	**0.046**	**0.034**	0.214	0.0356	**0.051**	**0.070**	0.204	0.0742
1.00_0.05	829	**0.053**	**0.033**	0.219	0.0351	**0.050**	**0.032**	0.215	0.0341	**0.050**	**0.072**	0.217	0.0755
0.95_0.35	1,011	**0.051**	**0.032**	0.195	0.0336	**0.041**	**0.032**	0.196	0.0334	**0.049**	**0.069**	0.200	0.0726
0.95_0.30	1,090	**0.052**	**0.032**	0.201	0.0338	**0.042**	**0.032**	0.201	0.0335	**0.049**	**0.068**	0.203	0.0717
0.95_0.25	1,406	**0.051**	**0.032**	0.201	0.0338	**0.041**	**0.032**	0.201	0.0336	**0.049**	**0.069**	0.209	0.0726
0.95_0.20	1,721	**0.052**	**0.033**	0.207	0.0346	**0.042**	**0.033**	0.207	0.0344	**0.049**	**0.069**	0.208	0.0729
0.95_0.15	2,099	**0.052**	**0.033**	0.207	0.0343	**0.042**	**0.033**	0.206	0.0340	**0.049**	**0.069**	0.209	0.0716
0.95_0.10	2,492	**0.052**	**0.033**	0.210	0.0344	**0.042**	**0.033**	0.209	0.0340	**0.050**	**0.070**	0.215	0.0715
0.95_0.05	3,029	**0.051**	**0.032**	0.211	0.0336	**0.040**	**0.031**	0.211	0.0324	**0.051**	**0.071**	0.224	0.0726
0.90_0.35	1,352	**0.053**	**0.033**	0.207	0.0351	**0.042**	**0.033**	0.208	0.0348	**0.049**	**0.070**	0.210	0.0738
0.90_0.30	1,777	**0.053**	**0.033**	0.208	0.0348	**0.042**	**0.033**	0.208	0.0345	**0.049**	**0.069**	0.208	0.0729
0.90_0.25	2,267	**0.052**	**0.033**	0.206	0.0347	**0.042**	**0.033**	0.206	0.0343	**0.048**	**0.069**	0.210	0.0713
0.90_0.20	2,753	**0.053**	**0.033**	0.211	0.0353	**0.043**	**0.034**	0.210	0.0350	**0.048**	**0.068**	0.207	0.0720
0.90_0.15	3,342	**0.053**	**0.033**	0.211	0.0350	**0.042**	**0.033**	0.210	0.0346	**0.048**	**0.068**	0.207	0.0721
0.90_0.10	3,884	**0.053**	**0.033**	0.212	0.0350	**0.043**	**0.033**	0.212	0.0346	**0.050**	**0.069**	0.215	0.0722
0.90_0.05	4,666	**0.052**	**0.032**	0.214	0.0342	**0.040**	**0.032**	0.215	0.0329	**0.050**	**0.070**	0.223	0.0732
0.85_0.35	1,630	**0.052**	**0.033**	0.206	0.0351	**0.041**	**0.033**	0.207	0.0348	**0.047**	**0.069**	0.208	0.0735
0.85_0.30	2,159	**0.052**	**0.033**	0.205	0.0348	**0.042**	**0.033**	0.205	0.0345	**0.047**	**0.068**	0.205	0.0729
0.85_0.25	2,748	**0.052**	**0.033**	0.205	0.0347	**0.041**	**0.033**	0.204	0.0344	**0.047**	**0.068**	0.208	0.0717
0.85_0.20	3,324	**0.053**	**0.033**	0.209	0.0353	**0.042**	**0.034**	0.209	0.0350	**0.047**	**0.068**	0.208	0.0722
0.85_0.15	4,022	**0.053**	**0.033**	0.209	0.0350	**0.042**	**0.033**	0.209	0.0346	**0.047**	**0.068**	0.206	0.0716
0.85_0.10	4,675	**0.053**	**0.033**	0.212	0.0350	**0.042**	**0.033**	0.212	0.0346	**0.049**	**0.069**	0.214	0.0712
0.85_0.05	5,569	**0.052**	**0.032**	0.213	0.0342	**0.040**	**0.032**	0.215	0.0329	**0.049**	**0.067**	0.223	0.0718
0.80_0.35	2,042	**0.052**	**0.033**	0.207	0.0352	**0.041**	**0.033**	0.208	0.0348	**0.047**	**0.068**	0.208	0.0705
0.80_0.30	2,675	**0.052**	**0.033**	0.208	0.0351	**0.041**	**0.033**	0.208	0.0347	**0.047**	**0.068**	0.207	0.0725
0.80_0.25	3,388	**0.052**	**0.033**	0.207	0.0351	**0.041**	**0.033**	0.207	0.0348	**0.047**	**0.068**	0.211	0.0716
0.80_0.20	4,088	**0.053**	**0.034**	0.211	0.0357	**0.042**	**0.034**	0.211	0.0354	**0.048**	**0.069**	0.212	0.0722
0.80_0.15	4,959	**0.053**	**0.034**	0.212	0.0354	**0.042**	**0.034**	0.212	0.0350	**0.048**	**0.068**	0.211	0.0717
0.80_0.10	5,762	**0.053**	**0.033**	0.214	0.0353	**0.042**	**0.033**	0.214	0.0348	**0.049**	**0.069**	0.217	0.0729
0.80_0.05	6,898	**0.052**	**0.033**	0.215	0.0344	**0.039**	**0.032**	0.218	0.0330	**0.050**	**0.070**	0.225	0.0735
**Average**		0.052	0.033	0.208	0.0350	0.042	0.033	0.208	0.034	0.049	0.069	0.210	0.073
***SD***		0.001	0.001	0.007	0.001	0.002	0.001	0.007	0.001	0.002	0.002	0.007	0.002
**CV%**		1.37	1.63	3.31	1.69	4.46	2.19	3.22	2.26	3.93	2.26	3.40	2.44

*Also shown are the regression slopes of the pairwise values between individuals on the logarithm of the spatial distance (*b*), the coefficient of determination (*R*^2^), and the *Sp-statistic*–a synthetic measure of spatial genetic structure (SGS) intensity. Bold values denote statistical significance at *p* < 0.05.*

**FIGURE 3:**
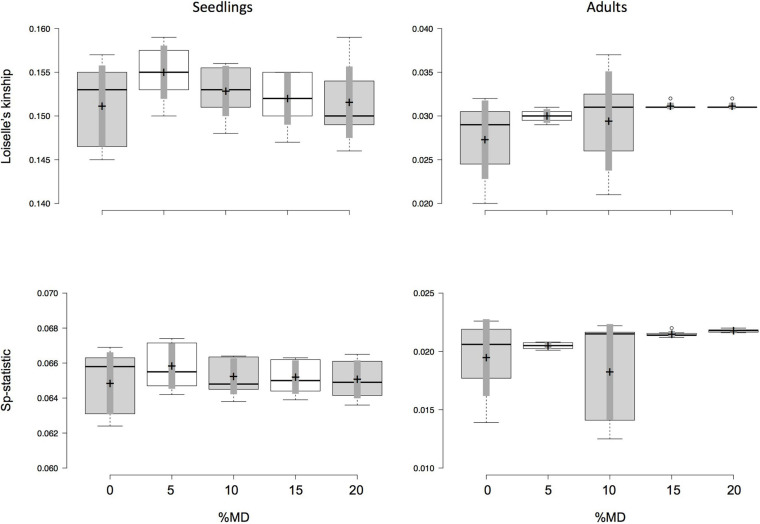
Effect of missing data (MD) and minor allele frequency (MAF) on values of Loiselle’s kinship measure for the first distance class (*F*_1_), which is less than 55 m between seedling and putative parent, and its derivative *Sp*-statistic for seedlings (left column) and adults (right column) of *Dinizia jueirana-facao*. The amount of MAF varied from 0.05 to 0.35 (i.e., seven data sets) for each fixed percentage of MD. Boxplots show the median (center line) and mean (marked by cross, with 95% confidence in gray) of *F*_1_ and *Sp*-statistic, and the 25th and 75th percentiles (by the box) with the extent of the whiskers marking 1.5 times the interquartile range from the 25th and 75th percentiles, and outliers (marked by dots), as calculated using R software.

Both seedlings and adult trees show a similar trend regarding the effects of MD and MAF on the fine-scale SGS estimates. However, estimates of *F*_1_ and *Sp*-statistic for data sets with different amounts of MD and MAF were more homogenous in seedlings than adult trees (Supplementary Table 2), with no statistical differences were observed among the data sets with the different amounts of MD and MAF (Figure 3). Note that no significant correlations between *F*_1_ or *Sp*-statistic and the number of SNPs were observed in either seedlings or adult trees (Supplementary Figure 1). On the other hand, the inbreeding coefficient estimates appear to be more sensitive to both MD and MAF, becoming inflated and statistical significant with increases of MD (Supplementary Figure 2 and Supplementary Table 3). For instance, with larger amounts of MD (with MAF varying from 0.05 to 0.35), the average inbreeding coefficient in seedlings of *D. jueirana-facao* increases and remains stastistical significant for data sets with 0 and 20% MD (e.g., 0% MD = 0.047, CI = 0.037 to 0.057, and 20% MD = 0.097, CI = 0.090 to 0.104; Supplementary Table 3). For adult trees of *D. jueirana-facao*, the average inbreeding coefficient was also statistically significant for 0% and 20% MD and shows similar trend (e.g., 0% MD = –0.065, CI = –0.075 to –0.055, and 20% MD = 0.005, CI = 0.001–0.008, Supplementary Table 3).

**FIGURE 4:**
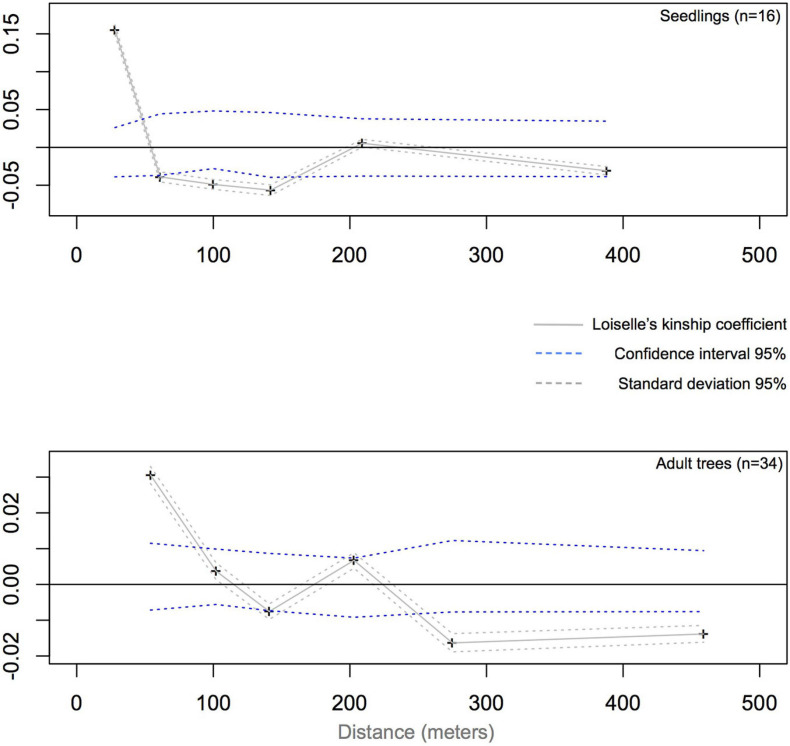
Average Loiselle’s kinship coefficient (*F*_L_) plotted against geographical distance (in gray solid line) between seedlings **(top plot)** and adults **(bottom plot)** of *Dinizia jueirana-facao.* Individuals whose standard deviation of kinship coefficient lie outside the 95% CIs (blue dashed lines) are significantly more similar than would be expected through random sampling. For both reproductive trees and seedlings, a decrease in the kinship values was detected with increasing distances, with significant negative values indicating that distant individuals are not genetically related. The results are based on analyses of a data set generated with 5% MD and a 5% minor allele frequency setting.

Based on distance class analysis, a significant linear decrease of the Loiselle’s kinship coefficients with the linear spatial distance was detected in both seedlings and adult trees (Figure 4). However, the shapes of the kinship curves are distinct for seedlings and adult trees (Figure 4), with significant positive values (based on the 95% CI) of up to 60 m for seedlings, and up to 100 m for adult trees. For seedlings, the largest Loiselle’s kinship coefficient (*F*_L_ = 0.155, *p* < 0.05) was estimated in the first class of distance (0–55 m). This value is between the theoretical expectation for half-siblings (*F*_L_ = 0.125) and full-sibs (*F*_L_ = 0.25). For adult trees, the largest kinship coefficient was observed in the first class of distance (*F*_L_ = 0.031, *p* < 0.05), a value consistent with expectations for second cousins (0.0312). Broadly speaking, stronger SGS was detected in seedlings compared to adult trees, as measured by *Sp*-statistic (*Sp* = 0.0651 in seedlings, *Sp* = 0.0208 in adults; Table 2).

**TABLE 2:** Estimates of fine-scale spatial genetic structure (SGS) and of historical dispersal distance for seedlings and adult tress of *Dinizia jueirana-facao*, as well as the inbreeding coefficient (*F*), the average kinship coefficient (*F*_1_) between individuals for the first distance class (i.e., the smallest distance class, which includes distances < 55 m) and its standard error (SE), the *Sp*-statistic, the neighborhood size, *Nb* and its 95% CI, and the root-mean-squared dispersal distance, σ and its 95% CI, are shown considering the effective densities of 0.237 (*D*_e_ = 0.79 × 0.30 for adult trees) and 0.111 (*D*_e_ = 0.37 × 0.30 for seedlings).

	SGS parameters				Gene dispersal estimates	
		
	*F*	*F*_1_	*SE*	*Sp*	*Nb*	σ (m)
Seedlings	**0.071**	**0.1553**	0.0032	0.0651	15.37 (15.2–15.4)	156.40 (155.8–156.9)
Adults	**–0.034**	**0.0306**	0.0012	0.0208	48.07 (47.9–48.2)	276.90 (276.3–276.9)

*Analyses are based on a data set with 5% of missing data and 0.05 of minor allele frequency. Bold values denote statistical significance at *p* < 0.05.*

The neighborhood size was estimated to be 15 individuals in the seedlings vs. 48 individuals in the adult trees’ generation. The average gene dispersal distances were estimated to be 156 m in the seedlings versus 277 m in the adult trees, with small confidence intervals for both (Table 2).

### Effects of MD and MAF on Parentage Analysis

The combined non-exclusion probabilities among parent pairs were extremely low, varying from 2.603^–227^ to 0.000 (Supplementary Table 4), irrespective of the MD and MAF settings. That is, the probability of cryptic gene flow was equal to zero for all but two data sets (Supplementary Table 4), indicating that there is no apparent sensitivity of parentage analysis with respect to the MD and MAF values for this empirical dataset.

In order to do comparisons with the indirect gene flow estimates, we also used the data set with 5% of MD and a MAF of 0.05 to quantify the direct gene flow in *D. jueirana-facao*. We assigned a maternal and paternal parent to 11 (69%) of the 16 seedlings with 95% confidence (Table 3). For the remaining five seedlings, a putative mother tree could be assigned. Among the 16 seedlings with assigned parentage, eight of the 34 (24%) reproductive trees in the RNV population were assigned as the maternal parent, and five of the 34 (15%) reproductive trees were assigned as the paternal parent; six seedlings were likely a product of selfing (*s* = 0.375). In total, only nine of 34 (26.5%) reproductive trees of *D. jueirana-facao* are parents of at least one seedling.

**TABLE 3:** Maximum-likelihood parentage analysis to assign maternal and paternal identities for 16 *Dinizia jueirana-facao* seedlings (i.e., the parents with the highest and second highest LOD score^1^; maternal and paternal individuals were identified by distance from the seedlings; see methods for details).

ID – Seedlings	ID – 1st parent	1st parent LOD score	ID – 2nd parent	2nd parent LOD score	Pair parent LOD score
pop1_102	**pop1_100**	8,56E + 15*	pop1_086	4,19E + 15*	1,79E + 16*
pop1_113	**pop1_111**	8,15E + 15*	pop1_114	8,86E + 15*	2,16E + 16*
pop1_012	pop1_001	3,41E + 15*	**pop1_008**	2,40E + 15*	8,37E + 15*
pop1_025	pop1_001	3,21E + 15*	**pop1_026**	5,42E + 14*	5,90E + 15*
pop1_027	pop1_024	-1,84E + 16	**pop1_026**	6,42E + 15*	0.00E + 00
pop1_028	**pop1_026**	7,49E + 15*	**pop1_026**	7,49E + 15*	1,42E + 16*
pop1_032	pop1_026	2,50E + 15*	**pop1_037**	2,16E + 15*	5,54E + 15*
pop1_034	**pop1_037**	4,23E + 15*	**pop1_037**	4,23E + 15*	8,23E + 15*
pop1_035	**pop1_037**	4,52E + 15*	**pop1_037**	4,52E + 15*	8,30E + 15*
pop1_036	**pop1_037**	4,60E + 15*	**pop1_037**	4,60E + 15*	5,51E + 15*
pop1_038	**pop1_037**	4,49E + 15*	**pop1_037**	4,49E + 15*	8,18E + 15*
pop1_039	**pop1_001**	7,58E + 15*	**pop1_001**	7,58E + 15*	1,46E + 16*
pop1_055	pop1_004	-1,83E + 16	**pop1_082**	2,00E + 16*	0.00E + 00
pop1_059	**pop1_037**	2,20E + 16*	pop1_004	-2,12E + 16	0.00E + 00
pop1_085	pop1_083	-8,31E + 15	**pop1_086**	1,98E + 16*	0.00E + 00
pop1_087	pop1_037	-5,04E + 15	**pop1_086**	2,51E + 15*	0.00E + 00

The sampling distribution is shown in Figure 1. ^1^LOD score is the natural logarithm of the likelihood that the candidate parent is the true parent divided by the likelihood that the candidate parent is not the true parent. *Delta criterion based on 95% confidence threshold from 50,000 simulations. Maternal parents are in bold.

The effective pollination distance was greater than the effective seed migration distance, with reproductive trees involved in pollination occurring 31.1–147.4 m (average of 73.80 ± 42.9 m) from the seedlings. The seed dispersal distance varied from 1.0 to 79.5 m, with an average of 19.11 ± 23.72 m. By taking into account the pollen and seed dispersal variances, the total direct gene flow distance of 41.8 m was 3.74-fold smaller than the one based on indirect estimates of gene flow for the seedlings (Table 2).

## Discussion

Our approach shows how different measures of gene flow (direct and indirect) are sensitive, or conversely robust, to the settings for the percentage of MD and MAF used in the bioinformatic processing of SNP data in a small plant population for which the very characteristics of being a small population, challenge the assumptions made in other studies investigating the robustness of gene flow measures to different settings used to filter genomic data. That is, although previous studies have investigated the effects of data filtering on SGS (Weir and Goudet, 2017; Attard et al., 2018; Goudet et al., 2018) and parentage analyses (e.g., Anderson and Garza, 2006; Baruch and Weller, 2008; Andrews et al., 2018; Dussault and Boulding, 2018; Hall et al., 2020), our study focuses on the combined effect of MD and MAF, which depending on the measure used to estimate gene flow, can introduce biases when applied to small populations specifically. Our approach is a general one that any researcher might apply to evaluate the potential sensitivity of their data set to MD and MAF settings so that they can accommodate this uncertainty into their analyses and interpretations of the results.

Given that estimates of gene flow have direct consequences for conservation decision on threatened species (e.g., Flanagan et al., 2018; Bowles et al., 2020), we reflect on our findings to emphasize that there is no ‘rule of thumb’ in the population genomic era (i.e., a universal set of settings for bioinformatic processing of genomic data). Instead, a sensitivity analysis such as the one applied here should be implemented to confirm how robust inferences might be for any particular study/data set with regards to different settings of MD and MAF (e.g., Catchen et al., 2011, 2013; Eaton, 2014). This methodological recommendation is essential to avoid unintentional biases that may result from applying particular filtering criteria to genomic data (e.g., Huang and Knowles, 2014; Hodel et al., 2017). Below, we discuss the implications of our results not only in light of general decisions regarding data filtering, but also on how our findings specifically can be useful for conservation strategies in the rare and critically endangered plant species *D. jueirana-facao*.

### Choosing MD and MAF Settings to Assess SGS and Parentage Analysis

The direct and indirect gene flow estimates in the *D. jueirana-facao* population showed little sensitivity to variations in MD and MAF. However, when more loci were used, we observed a decrease in the standard error for direct and indirect gene flow estimates (e.g., relatedness; Supplementary Table 1), indicating that the data set with more loci (i.e., more missing data permitted) is better suited to obtain population genetic parameters. Nevertheless, in all analyses we chose to use the data set with 5% MD and 0.05 MAF (3,029 SNPs) instead of 20% MD and 0.05 MAF (6,898 SNPs). This is due to the fact that estimates of inbreeding – a genetic parameter directly associated with estimates of relatedness (Cockerham, 1966) – were unbiased using the 0 or 5% MD data set (Supplementary Table 3), and the 5% MD and 0.05 MAF data set offered a greater number of loci than the 0% MD and 0.05 MAF data set.

### Guidelines, but Not a Rule of Thumb

Anonymous sequencing methods, such as the reduced representation of genomes protocols [e.g., Genotyping-By-Sequencing (GBS), restriction site-associated DNA sequencing (RAD-seq), double-digest RAD-seq (ddRADseq), and Complexity Reduction of Polymorphic Sequences (CRoPS), ezRAD, and CUTseq; see Andrews et al., 2016; Zhang et al., 2019 for more details], are commonly used to identify biallelic SNPs for a broad range of questions in a diversity of taxa. With this increased application of SNPs, there has been increasing attention being paid to the downstream effects of bioinformatic data processing (e.g., Huang and Knowles, 2014; Paris et al., 2017; Cumer et al., 2021). For example, artifacts generated during bioinformatic processing (or even during genomic library construction) can result in misleading biological conclusions (e.g., O’Leary et al., 2018; Larson et al., 2020), such as incorrect inferences about the geographic structure of genetic variation because of the settings used to filter genomic data (see Larson et al., 2020).

Both the molecular technology used to produce genetic data (e.g., RADseq, where the number of individuals used and size selection of fragments used to create the library, in conjunction with genome size of the species, influence the coverage and missing data), and the bioinformatic settings used to process the genomic data determines the properties of a data set (e.g., the amount of, and which data, are retained for analysis). For example, to estimate intra- and interpopulation genetic parameters, the MAF filter set as low as 1–5% (Rochette and Catchen, 2017) will help ensure that alleles will be found within each population. However, the MAF depends on the sample size, which means that even with a low setting for the MAF filter, inferences may not be robust (e.g., with five individuals sampled per locality, the MAF in the population is 10%). The evolutionary history of the species itself can also impact how many and which loci are retained when filtering the data. For example, the level of divergence among the sampled individuals is a reflection of the evolutionary context (e.g., persistent stable populations versus expanding ones). Likewise, the frequencies of SNPs vary as a function of the demographic history of the species (or populations; e.g., a population expansion versus subdivided population structure).

The desirable properties of a retained data set depends upon the application of the genomic data and the assumptions of the particular methods that will be used to analyze the data (Flanagan et al., 2018). For example, some applications of genomic data require especially high confidence in the calls of SNPs, whereas others do not (e.g., genome-wide association studies versus phylogenetic inference, respectively) because of differences in the relative sensitivity of an inference to errors in SNP-calling. Likewise, some analytical methods require no missing data. Other methods can accommodate missing data, but these methods vary in how more or less robust they are to missing data. Consequently, the inherent properties of data sets will be unique to each study, as will the level of uncertainty that can be accommodated for accurate inference. All of this means that there are no rules of thumb (i.e., a general suite of settings) for bioinformatic processing of genomic data. Instead, the filtering of genomic data will require data set specific settings, and depending on the analytical method being applied, different data sets may need to be generated from the same genomic data for any one particular study (e.g., Resende-Moreira et al., 2019; Massatti and Knowles, 2020; Marske et al., 2020).

All these considerations for exploring the sensitivity of inferences to the properties of data sets that are either intrinsic to the species evolutionary history itself, or are shaped by the bioinformatic processing of the genomic data, apply to the empirical data of *D. jueirana-facao* for inferences about its dispersal. For example, the sensitivity analyses indicated that the inbreeding coefficient was more sensitive to the percentage of MD than either the direct or indirect estimates of gene flow. As such, applying the arbitrary cutoff of MD (e.g., 20%) that is often applied (e.g., Catchen et al., 2013; Kang et al., 2017; Wyngaarden et al., 2017; Flanagan et al., 2018; Ríos et al., 2020; Soghigian et al., 2020), or even advised (see Catchen et al., 2013), would generate spurious results. Moreover, these different estimates would also change the interpretation and conclusions we might make about *D. jueirana-facao*. For instance, the average inbreeding coefficient in adult trees was statistically significant for a range of percent MD and ranged from –0.065 (for 0% MD) to 0.005 (for 20% MD). Because the interpretation of these statistic shifts when the inbreeding coefficient is negative or positive, an arbitrarily chosen 20% MD would have indicated a low, but significant, level of inbreeding in the adult trees of *D. jueirana-facao*. Does this mean that 20% should not be used in other studies? Absolutely not. Although the use of the full data set (i.e., no missing data) instead of 20% MD provided an unbiased estimate of the level of inbreeding in the trees of *D. jueirana-facao*, the use of 20% MD for other data sets may be a good setting, maximizing the number of loci without biasing the results (e.g., Hovmöller et al., 2013).

We observed that the sensitivity to MD varies among the summary statistics, as it does among studies. For example, Hodel et al. (2017) found that some, but not all, genetic estimates (e.g., *F*_IS_, *F*_ST_, and *H*_e_) were sensitive to the amount of MD (i.e., varied depending upon the amount of MD) in mangroves. Likewise, comparing simulated with empirical ddRAD data set, Attard et al. (2018) also reported that relatedness estimates, specifically that proposed by Ritland (1996), is robust to MD between 0 and 40%. We also found that there were no detectable effects of MD on the relatedness estimates (Table 1); however, our study differs in that Loiselle’s kinship, not Ritland’s kinship, is less sensitive to the MD, as well as the MAF setting used to filter the genomic data of *D. jueirana-facao*. This difference may relate to the intrinsic properties arising from the demographic history of *D. jueirana-*facao given that Ritland’s kinship estimator tends to give downward biased estimates when rare alleles occur (Ritland, 1996; Vekemans and Hardy, 2004).

### Does Different Filtering Setting per Study Confound Comparisons Across Studies?

The comparison across species in their genetic structuring is essential for exploring the generality of evolutionary hypotheses (e.g., the identification of shared effects of climate induced distributional shifts (e.g., Knowles et al., 2016; Myers et al., 2019). However, the different criteria for bioinformatic processing of genomic data across studies does not compromise comparisons across studies. As discussed above, standardizing these setting would introduce biases of varying degrees across studies; if the inferences for individuals studies are biased, there is no reasonable argument that standardization could improve the accuracy of conclusions drawn from comparing those studies. In addition, the bias introduced by applying a single standard across species would obscure any effort to quantify the uncertainty in estimating parameters or testing hypotheses of interspecific similarities or dissimilarities (e.g., Andrews et al., 2018; Díaz-Arce and Rodríguez-Ezpeleta, 2019).

Likewise, the view that since more stringent settings will reduce SNP-calling errors, these settings are desirable as being “more conservative” is inaccurate. For example, because the amount of data impacts both the accuracy and error associated with parameter estimates (e.g., Arnold et al., 2013; Marandel et al., 2020), the loss of information when applying strict filtering criteria that severely reduce the number of SNPs will not be outweighed by reducing potential SNP-calling errors (e.g., Mastretta-Yanes et al., 2015; Paris et al., 2017; Díaz-Arce and Rodríguez-Ezpeleta, 2019; Cumer et al., 2021).

### A Small, but Not Isolated Population and Implication for Conservation Management

The impact of the specific history of *D. jueirana-facao* – that is, a small plant population that resulted from habitat loss – appears to affect how generalizable previous suggestions about MD and MAF might be (e.g., Anderson and Garza, 2006; Baruch and Weller, 2008; Strucken et al., 2016; Andrews et al., 2018). Other studies have similarly documented different degrees of sensitivity, and they were not all based on small populations (e.g., Chattopadhyay et al., 2014; Hodel et al., 2017; Attard et al., 2018; Dussault and Boulding, 2018; O’Connell and Smith, 2018; Crotti et al., 2019; Díaz-Arce and Rodríguez-Ezpeleta, 2019; Larson et al., 2020; Cumer et al., 2021). Together, this reinforces that any general guideline for bioinformatic settings still need to be examined with sensitivity analyses on a case-by-case basis. Such study-specific and/or data set specific settings therefore also become an important part of investigating the crises many species face due to habitat loss and shrinking population sizes.

Our findings demonstrate the robustness of gene flow estimates, as well as the sensitivity of some summary statistics, and provide essential information about the uncertainty arising from the settings of MD and MAF used in the bioinformatic processing of the genomic data for *D. jueirana-facao*. This information for this small, fragmented population is vital to avoiding biased inferences about gene flow that inform conservation and management policies of *D. jueirana-facao*. In particular, with contemporary estimates of gene dispersal distance (σ*_r_*_t_ = 41.8 m) ∼ fourfold lower than the historical estimates, the genetic consequences of the recent restriction in the scale of gene flow identifies the magnitude of the threat posed by forest fragmentation and loss of habitat in *D. jueirana-facao*.

The response of different plant species with specific pollination or seed dispersal syndromes may vary when faced with anthropogenic disturbances (e.g., Bacles and Jump, 2011; Hardy et al., 2019). However, with respect to tree species, they exhibit a trend of reduced contemporary gene flow, even if they differ in their respective dispersal syndromes (e.g., Oddou-Muratorio and Klein, 2008; Guidugli et al., 2016; but see Bacles et al., 2005). However, the magnitude of the decrease of contemporary gene flow varied among tree species. For instance, a contemporary estimate of gene dispersal distance was twofold smaller than the historical estimates for the insect-pollinated and animal-dispersed tree species *Sorbus torminalis* (Oddou-Muratorio and Klein, 2008), whereas in the wind-dispersed tree species *Entandrophragma cylindricum* the reduction in contemporary gene flow was almost threefold compared with historical estimates of gene flow (Monthe et al., 2017).

With respect to the dispersal distance, contemporary versus historical measures differ in *D. jueirana-facao*. However, we note that realized gene dispersal distances are markedly higher for pollen than seeds, which is consistent with other studies (e.g., Oddou-Muratorio and Klein, 2008; Berens et al., 2013; Guidugli et al., 2016; Hardy et al., 2019). The effectiveness of pollen transport may be a key contributor to the resilience of *D. jueirana-facao* to losses related to anthropogenic threats, given that selfing rates estimated from parentage analysis were not exceedingly high. Predominantly outcrossing tree species such as *D. jueirana-facao*, like other species, are expected to show pollen dispersal over long distances (e.g., Bacles et al., 2005; Nazareno and Carvalho, 2009; Guidugli et al., 2016; Hardy et al., 2019). Indeed, even in the highly fragmented landscape that *D. jueirana-facao* inhabits, we observed a moderate frequency (31.25%) of gene immigration, indicating that pollen movement beyond the edges of the small fragment may reach distances of 12 km (i.e., there is long-distance pollen dispersal between the forest fragment and the nearest pollen source). Gene flow by pollen dispersal beyond the edges of seemingly isolated forest fragments has been documented for distinct tree species, including species that are animal-pollinated (e.g., Nason and Hamrick, 1997; Sato et al., 2006; Nazareno and Carvalho, 2009; Ottewell et al., 2012; Côrtes et al., 2013; Saro et al., 2014; Guidugli et al., 2016; Garcia et al., 2019; Skogen et al., 2019; Lompo et al., 2020). Our result suggests that the open landscape due to deforestation, where the small population of *D. jueirana-facao* are located, facilitates pollen flow and may ameliorate the expected detrimental genetic effects of forest fragmentation. In fact, the inbreeding rate in the seedlings of *D. jueirana-facao* was close to zero. However, the sustainability of the small number of individuals of the species in the long-term is unclear, given that the maintenance of gene flow depends on the preservation of very small populations of *D. jueirana-facao* that reside in forest remnants that are highly fragmented across the landscape.

Our estimates of indirect dispersal distance also provide direct practical guidance for the conservation of *D. jueirana-facao.* For example, our genomic study suggests that efforts toward managed reseeding programs should focus on collecting seeds for breeding, conservation, and restoration programs from reproductive trees separated by at least 100 m. This finding, along with the maximum estimate of direct gene dispersal observed within the population (∼275.0 m), should be taken into account in management strategies to promote more favorable conditions for the establishment and retention of new generations of seedlings. Furthermore, we noted that the population of *D. jueirana-facao* received a moderate percentage of long-distance immigrant pollen, indicating that this small population is not genetically isolated. This direct dispersal pattern is relevant for the *in situ* conservation of remaining local populations since gene flow over long distances can enhance and/or increase connectivity between the two remaining fragments of *D. jueirana-facao*. As such, any human activities that would jeopardize the connectivity between fragments, and in essence exacerbating the negative genetic effects of small, isolated populations (e.g., Spielman et al., 2004), would place the viability of forest remnants in immediate peril. Several strategies have proven to increase connectivity between fragments, notably the establishment of corridors along forest patches (e.g., Rosot et al., 2018), and increasing the porosity of the matrix (e.g., Rodrigues et al., 2009; Rubio and Saura, 2012). Such measures must be informed by empirical data in which the gene flow capacity of species is measured directly on the particular landscape associated with the species of interest to avoid applying generalities to manage fragmented plant populations, when these populations exhibit species-specific responses, as well as species-specific means for alleviating the negative genetic consequences of population loss and fragmentation.

## Data Availability Statement

SNP data sets for *D. jueirana-facao* are available for download from the Dryad Digital Repository (https://doi.org/10.5061/dryad.0vt4b8h01). The raw data generated for *D. jueirana-facao* are available to download from the ENA (European Nucleotide Archive) under accession number ERP129560.

## Author Contributions

AGN and LK designed the study. AGN collected the samples, conducted the molecular work, performed the analyses, and led the writing of the manuscript with input from LK, who also provided analytical support. Both authors contributed to the article and approved the submitted version.

## Conflict of Interest

The authors declare that the research was conducted in the absence of any commercial or financial relationships that could be construed as a potential conflict of interest.

## Publisher’s Note

All claims expressed in this article are solely those of the authors and do not necessarily represent those of their affiliated organizations, or those of the publisher, the editors and the reviewers. Any product that may be evaluated in this article, or claim that may be made by its manufacturer, is not guaranteed or endorsed by the publisher.

## Footnotes

1
https://CRAN.R-project.org/package=adegenet
