# Supplementary material for: There Is No ‘Rule of Thumb’: Genomic Filter Settings for a Small Plant Population to Obtain Unbiased Gene Flow Estimates
Source: Front Plant Sci. 2021 Oct 14;12:677009. doi: 10.3389/fpls.2021.677009 (PMC8551369; doi:10.3389/fpls.2021.677009)
Supplement: Supplementary file 1 [file Data_Sheet_1.docx]

**THERE IS NO ‘RULE OF THUMB’: GENOMIC FILTER SETTINGS FOR A SMALL PLANT POPULATION TO OBTAIN UNBIASED GENE FLOW ESTIMATES**

**Supporting Information**

Alison G. Nazareno^1,2^ , L. LACEY KNOWLES^1^

^1^ Department of Ecology and Evolutionary Biology, University of Michigan, Ann Arbor, MI, USA.

^2^ Department of Genetics, Ecology and Evolution, Federal University of Minas Gerais, Belo Horizonte, MG, Brazil.

**TABLE S1** Standard error (SE) of the pairwise relatedness statistics (*F_1_*) for the empirical data set (*n* = 50; adults and seedlings of *Dinizia jueirana-facao* combined) based on different amounts of missing data (MD) and minor allele frequency (MAF).

|  |  | Loiselle’s kinship | | Ritland’s kinship | | Q&G’s relatedness | |
| --- | --- | --- | --- | --- | --- | --- | --- |
| **MD_MAF** | **SNPs** | ***F_1_*** | **SE** | ***F_1_*** | **SE** | ***F_1_*** | **SE** |
| 0.80_0.05 | 6,898 | **0.052** | 0.0018 | **0.039** | 0.0086 | **0.050** | 0.0019 |
| 0.80_0.10 | 5,762 | **0.053** | 0.0011 | **0.042** | 0.0086 | **0.049** | 0.0021 |
| 0.80_0.15 | 4,959 | **0.053** | 0.0016 | **0.042** | 0.0094 | **0.048** | 0.0022 |
| 0.80_0.20 | 4,088 | **0.053** | 0.0016 | **0.042** | 0.0106 | **0.048** | 0.0023 |
| 0.80_0.25 | 3,388 | **0.052** | 0.0018 | **0.041** | 0.0116 | **0.047** | 0.0024 |
| 0.80_0.30 | 2,675 | **0.052** | 0.0020 | **0.041** | 0.0129 | **0.047** | 0.0026 |
| 0.80_0.35 | 2,042 | **0.052** | 0.0017 | **0.041** | 0.0147 | **0.047** | 0.0030 |
| 0.85_0.05 | 5,569 | **0.052** | 0.0011 | **0.040** | 0.0090 | **0.049** | 0.0023 |
| 0.85_0.10 | 4,675 | **0.053** | 0.0014 | **0.042** | 0.0089 | **0.049** | 0.0024 |
| 0.85_0.15 | 4,022 | **0.053** | 0.0018 | **0.042** | 0.0096 | **0.047** | 0.0024 |
| 0.85_0.20 | 3,324 | **0.053** | 0.0020 | **0.042** | 0.0109 | **0.047** | 0.0025 |
| 0.85_0.25 | 2,748 | **0.052** | 0.0020 | **0.041** | 0.0119 | **0.047** | 0.0027 |
| 0.85_0.30 | 2,159 | **0.052** | 0.0019 | **0.042** | 0.0132 | **0.047** | 0.0029 |
| 0.85_0.35 | 1,630 | **0.052** | 0.0023 | **0.041** | 0.0150 | **0.047** | 0.0034 |
| 0.90_0.05 | 4,666 | **0.052** | 0.0022 | **0.040** | 0.0093 | **0.050** | 0.0025 |
| 0.90_0.10 | 3,884 | **0.053** | 0.0020 | **0.043** | 0.0092 | **0.050** | 0.0027 |
| 0.90_0.15 | 3,342 | **0.053** | 0.0025 | **0.042** | 0.0099 | **0.048** | 0.0026 |
| 0.90_0.20 | 2,753 | **0.053** | 0.0027 | **0.043** | 0.0110 | **0.048** | 0.0027 |
| 0.90_0.25 | 2,267 | **0.052** | 0.0025 | **0.042** | 0,0120 | **0.048** | 0.0029 |
| 0.90_0.30 | 1,777 | **0.053** | 0.0028 | **0.042** | 0.0133 | **0.049** | 0.0032 |
| 0.90_0.35 | 1,352 | **0.053** | 0.0028 | **0.042** | 0.0152 | **0.049** | 0.0037 |
| 0.95_0.05 | 3,029 | **0.051** | 0.0027 | **0.040** | 0.0110 | **0.051** | 0.0032 |
| 0.95_0.10 | 2,492 | **0.052** | 0.0022 | **0.042** | 0.0105 | **0.050** | 0.0032 |
| 0.95_0.15 | 2,099 | **0.052** | 0.0031 | **0.042** | 0.0113 | **0.049** | 0.0033 |
| 0.95_0.20 | 1,721 | **0.052** | 0.0030 | **0.042** | 0.0126 | **0.049** | 0.0035 |
| 0.95_0.25 | 1,406 | **0.051** | 0.0027 | **0.041** | 0.0140 | **0.049** | 0.0037 |
| 0.95_0.30 | 1,090 | **0.052** | 0.0030 | **0.042** | 0.0156 | **0.049** | 0.0041 |
| 0.95_0.35 | 1,011 | **0.051** | 0.0035 | **0.041** | 0.0178 | **0.049** | 0.0048 |
| 1.00_0.05 | 829 | **0.053** | 0.0032 | **0.050** | 0.0199 | **0.050** | 0.0057 |
| 1.00_0.10 | 778 | **0.054** | 0.0038 | **0.046** | 0.0187 | **0.051** | 0.0058 |
| 1.00_0.15 | 668 | **0.053** | 0.0037 | **0.044** | 0.0199 | **0.052** | 0.0059 |
| 1.00_0.20 | 537 | **0.053** | 0.0041 | **0.043** | 0.0255 | **0.052** | 0.0062 |
| 1.00_0.25 | 456 | **0.052** | 0.0040 | **0.041** | 0.0235 | **0.053** | 0.0066 |
| 1.00_0.30 | 338 | **0.053** | 0.0044 | **0.043** | 0.0275 | **0.054** | 0.0076 |
| 1.00_0.35 | 256 | **0.054** | 0.0053 | **0.044** | 0.0309 | **0.055** | 0.0088 |

Q&G - Queller & Goodnight’s relatedness

**TABLE S2** Estimates of different pairwise statistics (*F_1_*) to test the intensity of SGS (*Sp* statistic) when applied across datasets with distinct percentange of missing data (MD) and minor allele frequency (MAF) for seedlings (n=16) and adult trees (n=34) of *D. jueirana-facao* sampled in a small population in the Atlantic rainforest (Espírito Santo State, Southeast Brazil). The regression slopes of the pairwise values between individuals on the logarithm of the spatial distance (*b*) and the coefficient of determination (*R*^2^) are also showed. Bold values denotate statistical significance at *p* < 0.05.

**TABLE S3** Average of the inbreeding coefficient estimated for adults and seedlings of *Dinizia jueirana-facao*. The amount of minor allele frequency varied from 0.05 to 0.35 (i.e., seven data sets) for each fixed percentage of missing data. The lower (-) and upper (+) values of the 95% confidence interval (CI) are also showed.

|  | Percentage of missing data | | | | |
| --- | --- | --- | --- | --- | --- |
| **Adults** | **0%** | **5%** | **10%** | **15%** | **20%** |
| Mean | -0.065 | -0.047 | -0.015 | -0.013 | 0.005 |
| 95%CI- | -0.075 | -0.055 | -0.024 | -0.019 | 0.001 |
| 95%CI+ | -0.054 | -0.040 | -0.007 | -0.007 | 0.008 |
|  |  |  |  |  |  |
| **Seedlings** |  |  |  |  |  |
| Mean | 0.047 | 0.064 | 0.076 | 0.085 | 0.097 |
| 95%CI- | 0.037 | 0.056 | 0.069 | 0.078 | 0.090 |
| 95%CI+ | 0.057 | 0.072 | 0.083 | 0.091 | 0.104 |

**TABLE S4** Non-exclusion probabilities and cryptic gene flow (*C_gf_*) calculated for the 35 data sets with percentage of missing data (MD) varying from 0 to 20, and minor allele frequency (MAF) varying from 0.05 to 0.35.

|  |  | Non-exclusion probabilities | | | *C_gf_* |
| --- | --- | --- | --- | --- | --- |
| MD_MAF | SNPs | First parent | Second parent | Parent pair | Parent Pair |
| 0.80_0.05 | 6,898 | 4.46269E-61 | 0.00000E+00 | 0.00000E+00 | 0.000000 |
| 0.80_0.10 | 5,762 | 1.6674E-190 | 0.00000E+00 | 0.00000E+00 | 0.000000 |
| 0.80_0.15 | 4,959 | 7.1932E-172 | 0.00000E+00 | 0.00000E+00 | 0.000000 |
| 0.80_0.20 | 4,088 | 2.09162E-80 | 0.00000E+00 | 0.00000E+00 | 0.000000 |
| 0.80_0.25 | 3,388 | 3.1424E-163 | 8.9197E-123 | 0.00000E+00 | 0.000000 |
| 0.80_0.30 | 2,675 | 1.1504E-131 | 5.3405E-231 | 0.00000E+00 | 0.000000 |
| 0.80_0.35 | 2,042 | 1.09423E-79 | 5.0751E-168 | 2.60370E-227 | 0.000000 |
| 0.85_0.05 | 5,569 | 2.44528E-93 | 0.00000E+00 | 0.00000E+00 | 0.000000 |
| 0.85_0.10 | 4,675 | 4.6313E-132 | 0.00000E+00 | 0.00000E+00 | 0.000000 |
| 0.85_0.15 | 4,022 | 6.65195E-19 | 1.7375E-250 | 0.00000E+00 | 0.000000 |
| 0.85_0.20 | 3,324 | 1.11774E-26 | 4.8648E-247 | 0.00000E+00 | 0.000000 |
| 0.85_0.25 | 2,748 | 1.4696E-121 | 8.77162E-46 | 0.00000E+00 | 0.000000 |
| 0.85_0.30 | 2,159 | 3.68944E-89 | 3.9720E-129 | 1.85635E-268 | 0.000000 |
| 0.85_0.35 | 1,630 | 3.65143E-17 | 7.15828E-40 | 9.4122E-175 | 0.000000 |
| 0.90_0.05 | 4,666 | 4.86531E-16 | 9.9300E-219 | 0.00000E+00 | 0.000000 |
| 0.90_0.10 | 3,884 | 2.65089E-80 | 3.4105E-193 | 0.00000E+00 | 0.000000 |
| 0.90_0.15 | 3,342 | 6.46345E-91 | 7.2081E-154 | 0.00000E+00 | 0.000000 |
| 0.90_0.20 | 2,753 | 9.18092E-28 | 3.0204E-212 | 0.00000E+00 | 0.000000 |
| 0.90_0.25 | 2,267 | 4.11699E-93 | 7.85695E-81 | 2.12220E-50 | 0.000000 |
| 0.90_0.30 | 1,777 | 5.26065E-34 | 6.90316E-91 | 4.4037E-136 | 0.000000 |
| 0.90_0.35 | 1,352 | 6.25137E-27 | 2.12763E-96 | 3.3850E-167 | 0.000000 |
| 0.95_0.05 | 3,029 | 5.22716E-20 | 4.3591E-102 | 3.3447E-269 | 0.000000 |
| 0.95_0.10 | 2,492 | 1.01595E-35 | 1.99866E-93 | 8.4353E-164 | 0.000000 |
| 0.95_0.15 | 2,099 | 1.32131E-52 | 2.3870E-103 | 2.21850E-62 | 0.000000 |
| 0.95_0.20 | 1,721 | 1.20447E-22 | 4.6674E-129 | 7.6033E-110 | 0.000000 |
| 0.95_0.25 | 1,406 | 1.38009E-01 | 7.12228E-58 | 9.89060E-05 | 0.003357 |
| 0.95_0.30 | 1,090 | 6.55762E-47 | 2.75440E-87 | 2.53762E-70 | 0.000000 |
| 0.95_0.35 | 1,011 | 2.65737E-40 | 1.16347E-68 | 2.20086E-51 | 0.000000 |
| 1.00_0.05 | 829 | 4.80088E-22 | 1.51347E-08 | 1.38559E-71 | 0.000000 |
| 1.00_0.10 | 778 | 5.16531E-20 | 1.09240E-12 | 2.22261E-61 | 0.000000 |
| 1.00_0.15 | 668 | 2.76967E-15 | 7.14426E-38 | 3.49586E-69 | 0.000000 |
| 1.00_0.20 | 537 | 2.02754E-14 | 8.32201E-36 | 2.88723E-07 | 0.000010 |
| 1.00_0.25 | 456 | 2.49359E-13 | 2.81823E-04 | 2.55243E-62 | 0.000000 |
| 1.00_0.30 | 338 | 6.35440E-13 | 1.86471E-27 | 5.40933E-20 | 0.000000 |
| 1.00_0.35 | 256 | 4.92149E-02 | 4.06361E-11 | 4.71554E-05 | 0.001602 |

**FIGURE S1** Pearson's correlation coefficient (*r*) between Loiselle’s kinship in the first distance class (*F*_1_), and its derivated *Sp* statistic, and number os SNPs as a direct function of the percentage of missing data and minor allele frequency.

**FIGURE S2** Effects of missing data (MD) and minor allele frequency (MAF) on inbreeding coefficient for adults and seedlings of *Dinizia jueirana-facao*.
